# Genetic dissection of the eggplant rhizosphere microbiome enables microbiome-assisted breeding

**DOI:** 10.1093/hr/uhag163

**Published:** 2026-04-23

**Authors:** Yarong Li, Weiliu Li, Guiyun Gan, Xiaofang Wang, Yaoyao Wu, Yangchun Xu, Alexandre Jousset, Yikui Wang, Qirong Shen, Gaofei Jiang, Zhong Wei

**Affiliations:** Jiangsu Provincial Key Lab for Solid Organic Waste Utilization, Key Lab of Organic-Based Fertilizers of China, Jiangsu Collaborative Innovation Center for Solid Organic Wastes, Educational Ministry Engineering Center of Resource-Saving Fertilizers, Nanjing Agricultural University, Nanjing, China; Vegetable Research Institute, Guangxi Academy of Agricultural Sciences, Nanning 530007, China; Vegetable Research Institute, Guangxi Academy of Agricultural Sciences, Nanning 530007, China; Jiangsu Provincial Key Lab for Solid Organic Waste Utilization, Key Lab of Organic-Based Fertilizers of China, Jiangsu Collaborative Innovation Center for Solid Organic Wastes, Educational Ministry Engineering Center of Resource-Saving Fertilizers, Nanjing Agricultural University, Nanjing, China; State Key Laboratory of Crop Genetics and Germplasm Enhancement, Nanjing Agricultural University, Nanjing 211800, China; Jiangsu Provincial Key Lab for Solid Organic Waste Utilization, Key Lab of Organic-Based Fertilizers of China, Jiangsu Collaborative Innovation Center for Solid Organic Wastes, Educational Ministry Engineering Center of Resource-Saving Fertilizers, Nanjing Agricultural University, Nanjing, China; Jiangsu Provincial Key Lab for Solid Organic Waste Utilization, Key Lab of Organic-Based Fertilizers of China, Jiangsu Collaborative Innovation Center for Solid Organic Wastes, Educational Ministry Engineering Center of Resource-Saving Fertilizers, Nanjing Agricultural University, Nanjing, China; Vegetable Research Institute, Guangxi Academy of Agricultural Sciences, Nanning 530007, China; Jiangsu Provincial Key Lab for Solid Organic Waste Utilization, Key Lab of Organic-Based Fertilizers of China, Jiangsu Collaborative Innovation Center for Solid Organic Wastes, Educational Ministry Engineering Center of Resource-Saving Fertilizers, Nanjing Agricultural University, Nanjing, China; Jiangsu Provincial Key Lab for Solid Organic Waste Utilization, Key Lab of Organic-Based Fertilizers of China, Jiangsu Collaborative Innovation Center for Solid Organic Wastes, Educational Ministry Engineering Center of Resource-Saving Fertilizers, Nanjing Agricultural University, Nanjing, China; Vegetable Research Institute, Guangxi Academy of Agricultural Sciences, Nanning 530007, China; Jiangsu Provincial Key Lab for Solid Organic Waste Utilization, Key Lab of Organic-Based Fertilizers of China, Jiangsu Collaborative Innovation Center for Solid Organic Wastes, Educational Ministry Engineering Center of Resource-Saving Fertilizers, Nanjing Agricultural University, Nanjing, China

## Abstract

Rhizosphere microbiome critically influences plant growth and health, yet the genetic mechanisms underlying host regulation of microbiome composition remain unclear. Here, we analyzed whole-genome genotype and 16S rhizosphere microbiome data from 432 globally sourced eggplant accessions (*Solanum melongena* L.). Eggplant population structure corresponded to geographic origins, with rhizosphere microbiomes varying significantly among subpopulations. Host genetics explains 9%–39% of the variation in individual microbial taxa abundance, with core taxa more affected by host genetic variation. Microbial genome-wide association studies (mGWAS) identified 1235 significant genetic variants associated with 46 core microbial taxa, revealing key regulatory loci including *chr10:7799021* near *MYB113* (associated with *Stenotrophomonas*, *P* = 2.16 × 10^−15^) and *chr10:19786889* near *BLH9* (associated with *Mycobacterium*, *P* = 1.24 × 10^−12^), as well as a chromosome 5 locus with specific regulatory effects on *Rhizobiales*. These microbiome-associated genetic variants were enriched in secondary metabolic pathways, including anthocyanin biosynthesis, benzoxazinoid biosynthesis, and brassinosteroid biosynthesis, indicating that hosts regulate microbial communities through complex metabolic networks. Notably, genetic loci controlling microbial community structure underwent strong directional selection across eggplant subpopulations from different geographic origins, providing evidence for host–microbe coadaptive evolution. This study elucidates genetic regulatory patterns of eggplant rhizosphere microbiomes, enriching the theoretical framework of plant–microbe coevolution, with broad implications for microbiome-assisted crop improvement and sustainable agriculture.

## Introduction

Rhizosphere microorganisms, core components of the plant holobiont, play crucial roles in plant nutrient uptake, pathogen defense, and stress responses [1]. In view of climate change and population growth, achieving precise regulation of agricultural microbiomes will prove essential for food security and agricultural sustainability [2]. It is herein imperative to begin to decipher the mechanisms that underlie microbial community assembly in order to realize this goal. The rhizosphere environment has been established as the primary driver of microbial community dynamics [3, 4], while accumulating evidence demonstrates that host genetic variation also shapes microbial community composition [5]. For instance, in tomato, the ripening inhibitor (RIN) transcription factor regulates fruit development and root exudate composition, thus selectively recruiting beneficial microbes to maintain plant health [6], the maize root development genes *LRT1* and *RUM1* influence rhizosphere microbial community assembly through altering root architecture [7], and rice use the nitrate transporter gene *NRT1.1B* to regulate nitrogen-transforming microbes in the rhizosphere [8]. However, most of these studies have focused on the functional characterization of individual genes, which shape microbial communities. In contrast, host traits tightly associated with microorganisms, such as immune responses [9, 10] and root architecture [11], are typically controlled by multiple genes acting collectively, limiting comprehensive understanding of the genetic mechanisms underlying plant–microbe interaction networks. Genome-wide association studies (GWAS) offer a way to help solve this problem [12]. Starting with the seminal 2014 study that first treated Arabidopsis rhizosphere microbial features as quantitative traits [13], microbial genome-wide association studies (mGWAS) have been progressively extended to major crops, including rice [14], maize [15], and sorghum [16]. Particularly in sorghum, the rhizosphere has been recognized as the most promising compartment for elucidating the genetic basis of host–microbe interactions [16].

Eggplant (*Solanum melongena* L.) is an important solanaceous vegetable crop in the developing world. It is a good source of vitamins, phenolics, and antioxidants, with high nutritional value [17]. During its long-term domestication and cultivation, eggplant has accumulated a large amount of germplasm resources and rich phenotypic diversity, which provides a valuable gene pool for germplasm innovation, genetic improvement, and variety breeding [18]. Studies have shown that crop domestication not only shapes plant phenotypes but also profoundly influences plant–microbe interactions [19]. As eggplant spread from its Southeast Asian center of origin around the globe and faced selective pressures from varying geographical regions and climatic conditions, complex coadaptive relationships may have developed between host genetic variation and microbial community assembly. Our preliminary research has revealed significant differences in rhizosphere microbial community composition between different varieties of eggplant [20]. Specific eggplant genotypes have been demonstrated to suppress soil-borne pathogens such as bacterial wilt through selective recruitment of beneficial rhizosphere bacteria [21], highlighting the close association between host genetics and rhizosphere microbial communities. Despite these pieces of knowledge, much remains to be discovered regarding the genetic basis of eggplant–microbe associations. A systematic elucidation of these genetic associations is important to discover key regulatory genes underlying host–microbe interactions, providing an important foundation for using microbiome-assisted approaches to breed eggplant crops.

We collected 432 eggplant cultivars from diverse geographic regions across the globe and exposed them to the same environment to grow under the same conditions for whole-genome resequencing and rhizosphere microbial 16S rRNA amplicon sequencing. We systematically characterized the genetic diversity among eggplant cultivars, as well as the variation in composition and structure of the rhizosphere microbiota across the population. The extent of host genetic influence on rhizosphere microbial assembly was quantified, and mGWAS were employed to identify candidate loci associated with the relative abundance or presence/absence of specific microbial taxa. We report genome-wide associations between eggplant genetic variation and rhizosphere microbiome composition, implicating plant secondary metabolic as the primary genetic levers of microbiome assembly. We further demonstrate that these loci underwent directional selection during geographic dispersal, revealing that plant–microbiome configurations were coadaptive outcomes of crop domestication. These findings establish a genetic framework for microbiome-assisted breeding in eggplant and illustrate a broadly applicable strategy: identifying heritable microbiome taxa, mapping the plant loci that control them, and engineer favorable plant–microbiome partnerships.

## Results

### Genomic variation in domesticated eggplant accessions

**Figure 1:**
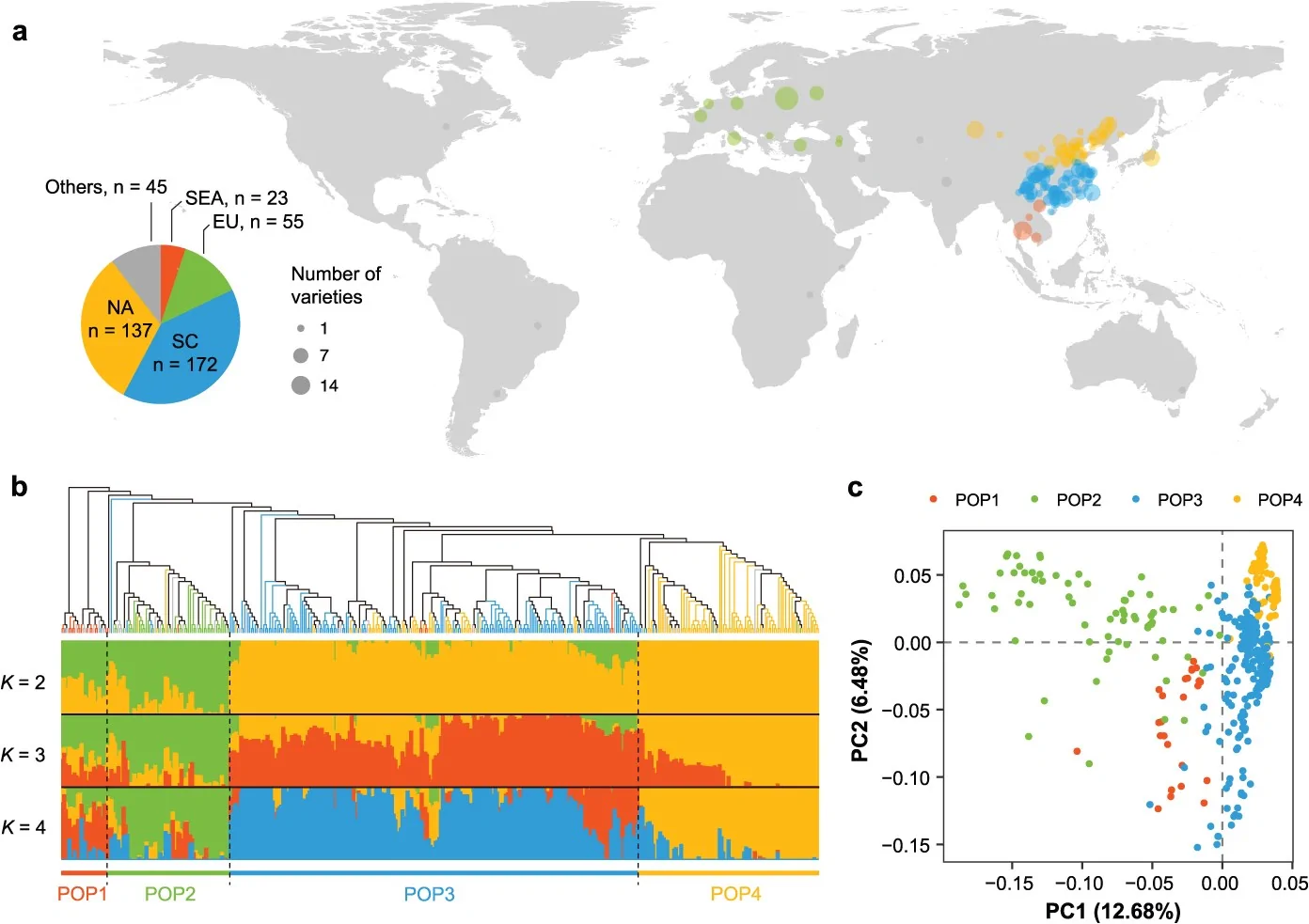
Geographic origins and population structure of 432 domesticated eggplant accessions. (a) Global geographical distribution of eggplant accessions in this study. Colors indicate geographic origins: Southeast Asia (SEA), Europe (EU), South China (SC), Northern Asia (NA), and other regions. Point size corresponds to variety count per region. (b) Phylogenetic tree and population structure (K = 2, 3, and 4) based on SNPs. Population structuring matches phylogenetic relationships. Tree colors correspond to geographic origins, while structure plot colors represent the genetic component state in each variety. (c) PCA based on SNPs, showing the first two principal components. Point colors correspond to different subpopulations.

The study included 432 domesticated eggplant accessions primarily originated from Southeast Asia (SEA, *n* = 23), Europe (EU, *n* = 55), South China (SC, *n* = 172), and Northern Asia (NA, *n* = 137, including North China and Japan) (Fig. 1a, Supplementary Table 1). Following whole-genome resequencing and stringent quality control, we identified 3 689 651 high-quality single nucleotide polymorphisms (SNPs, Supplementary Table 2). These SNPs were evenly distributed across chromosomes (3.29 SNPs/kb), with linkage disequilibrium (LD) decay distance of 1.06 Mb (Supplementary Fig. 1). The SNP-based phylogenetic tree divided all accessions into four subpopulations that corresponded well with their geographic origins (Fig. 1b, Supplementary Table 1). POP1 primarily comprised accessions from Southeast Asia (15/26, 57.7%). POP2 was predominantly European (48/70, 68.6%). POP3 mainly consisted of accessions from southern China, including Sichuan, Guangdong, Guangxi, and Yunnan provinces (160/233, 68.7%), while POP4 was dominated by accessions from northern China, including Shandong, Liaoning, Heilongjiang, and Hebei provinces (85/103, 82.5%). Population structure analysis further revealed the genetic structure at different K values (Fig. 1b). At K = 4, the division of subpopulations was consistent with their geographical origin and phylogenetic relationships. Principal component analysis (PCA) confirmed these findings, with PC1 and PC2 explaining 12.68% and 6.48% of genetic variance, respectively (Fig. 1c). Subpopulations exhibited substantial variation in nucleotide diversity (*π* = 0.17–0.28) and genetic differentiation (*F_ST_* = 0.13–0.28) (Supplementary Table 3). POP1 and POP2 showed higher diversity and closer genetic relationships, whereas POP4 was highly differentiated from them. This genetic variation, shaped by geographic isolation and environmental adaptation, provided an ideal foundation to investigate the regulatory role of host genotype on rhizosphere microbiomes.

### Rhizosphere microbiome composition across different subpopulations

**Figure 2:**
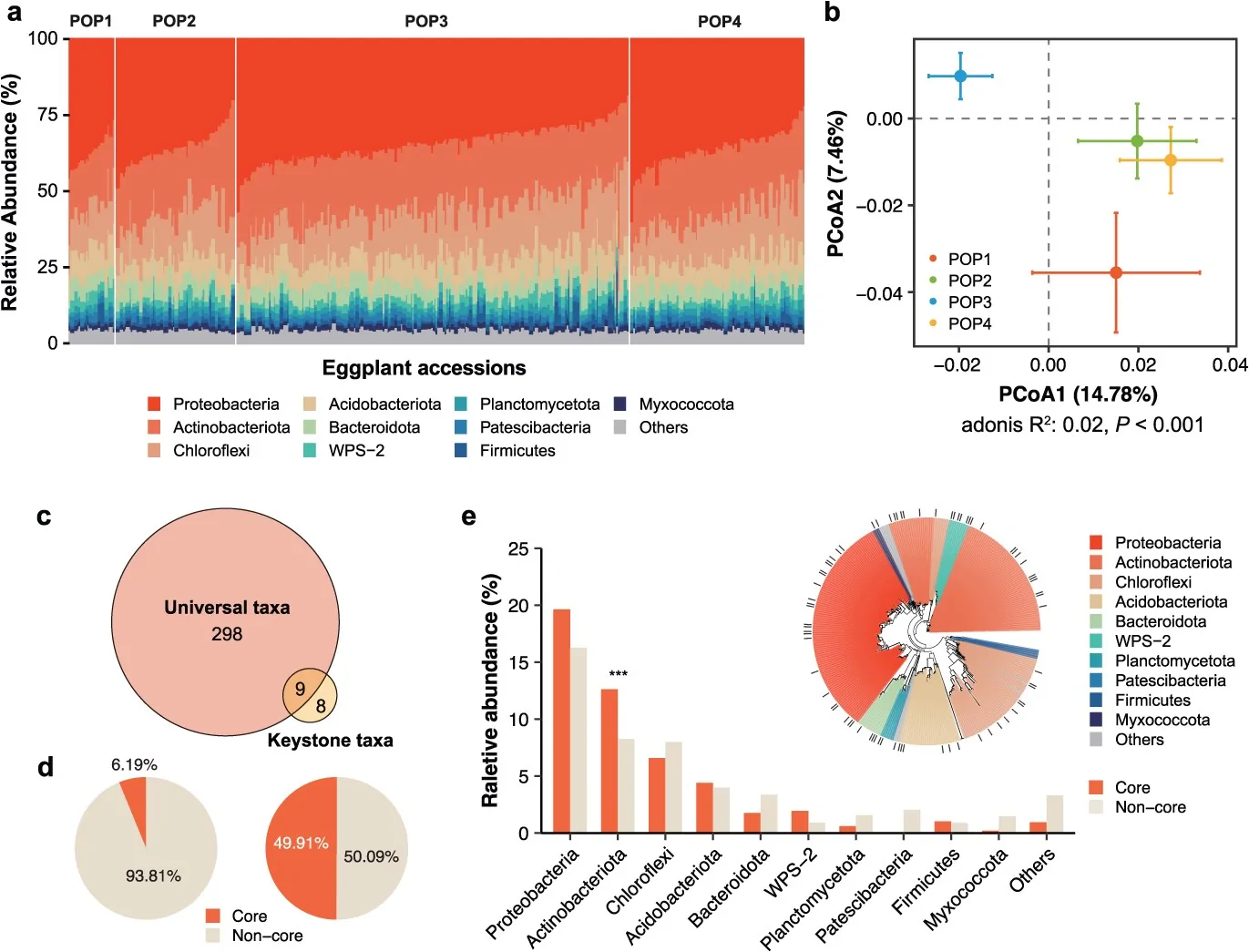
Core microbiome identification and composition in 432 eggplant accessions. (a) Rhizosphere microbial composition of eggplant accessions. Top 10 phyla in the rhizosphere are displayed. (b) PCoA of rhizosphere microbiome. Point colors correspond to different subpopulations. (c) Venn diagram illustrating the union of two approaches for defining core taxa: universal taxa (100% prevalence) and keystone taxa (identified by Zi-Pi topological analysis). Integration of both methods identified 315 core OTUs. (d) Pie charts comparing the proportion of core vs noncore taxa by OTU number (left) and relative abundance (right). (e) Relative abundance of core microbiome at the phylum level. Core microbiome was significantly enriched in Actinobacteriota (Fisher’s exact test, *P* < .05). Accompanying phylogenetic tree depicts 315 core OTUs, with the outer bars indicating core heritable taxa.

To investigate the impact of host genotype on rhizosphere microbial communities, we characterized the bacterial composition of 432 eggplant accessions using 16S rRNA gene sequencing. Operational taxonomic unit (OTU) clustering based on 97% sequence similarity resulted in 181 528 OTUs. To eliminate sequencing depth bias, 158 318 OTUs were retained after rarefaction. For downstream analyses, we focused on prevalent OTUs (*n* = 5091) detected in >20% of samples, which collectively accounted for 82.4% of total reads. Taxonomic annotation encompassed 26 phyla, 63 classes, 138 orders, 183 families, 345 genera, and 170 species. At the phylum level, the rhizosphere microbiome was dominated by Proteobacteria (35.8%), Actinobacteriota (21.1%), Chloroflexi (17.8%), and Acidobacteriota (8.5%) (Fig. 2a). Notably, Actinobacteriota exhibited similar relative abundance across POP2, POP3, and POP4, but was significantly depleted in POP1. Conversely, Acidobacteriota and WPS-2 were significantly enriched in POP1 than other subpopulations (Supplementary Fig. 2a, analysis of variance (ANOVA), *P* < .05). POP4 showed higher microbiome richness and Shannon diversity than POP3 (Supplementary Fig. 2b; ANOVA, *P* < .05). Principal coordinate analysis (PCoA) demonstrated distinct clustering of microbial communities among subpopulations (Fig. 2b; *R*^2^ = 0.02, *P* < .001). These differences indicate that the four subpopulations defined by host genetics exhibit distinct rhizosphere microbial compositions and community structures, supporting an association between host genetics and rhizosphere microbiome variation.

We employed two approaches to define the core rhizosphere microbiome, aiming to identify microbial taxa with high stability and ecological importance in the eggplant rhizosphere. First, using a prevalence-based approach, we defined universal taxa as OTUs detected in 100% of samples, identifying 307 OTUs (Supplementary Fig. 3a and Supplementary Table 4). Meanwhile, following the method proposed by Guimerà and Amaral [22], we identified keystone taxa by calculating within-module connectivity (*Zi*) and among-module connectivity (*Pi*) for each node in the microbial co-occurrence network, resulting in 17 OTUs (Supplementary Fig. 3b and c and Supplementary Table 5). Integration of both approaches yielded 315 core OTUs (Fig. 2c), representing 6.19% of all OTUs but accounting for 49.91% of the total microbial community in relative abundance (Fig. 2d), indicating that minority core taxa contribute the majority of community abundance. The core microbiome was dominated by Proteobacteria, Actinobacteriota, and Chloroflexi, which collectively comprised 77.90% of the core microbiome (Fig. 2e). Moreover, the core microbiome was highly correlated with the total microbiome (Mantel test, *r* = 0.91, *P* < .001), demonstrating that despite representing only 6.19% of total OTUs, core taxa effectively capture the overall ecological characteristics of the rhizosphere microbial community.

### Heritability of rhizosphere microbial features in eggplant

**Figure 3:**
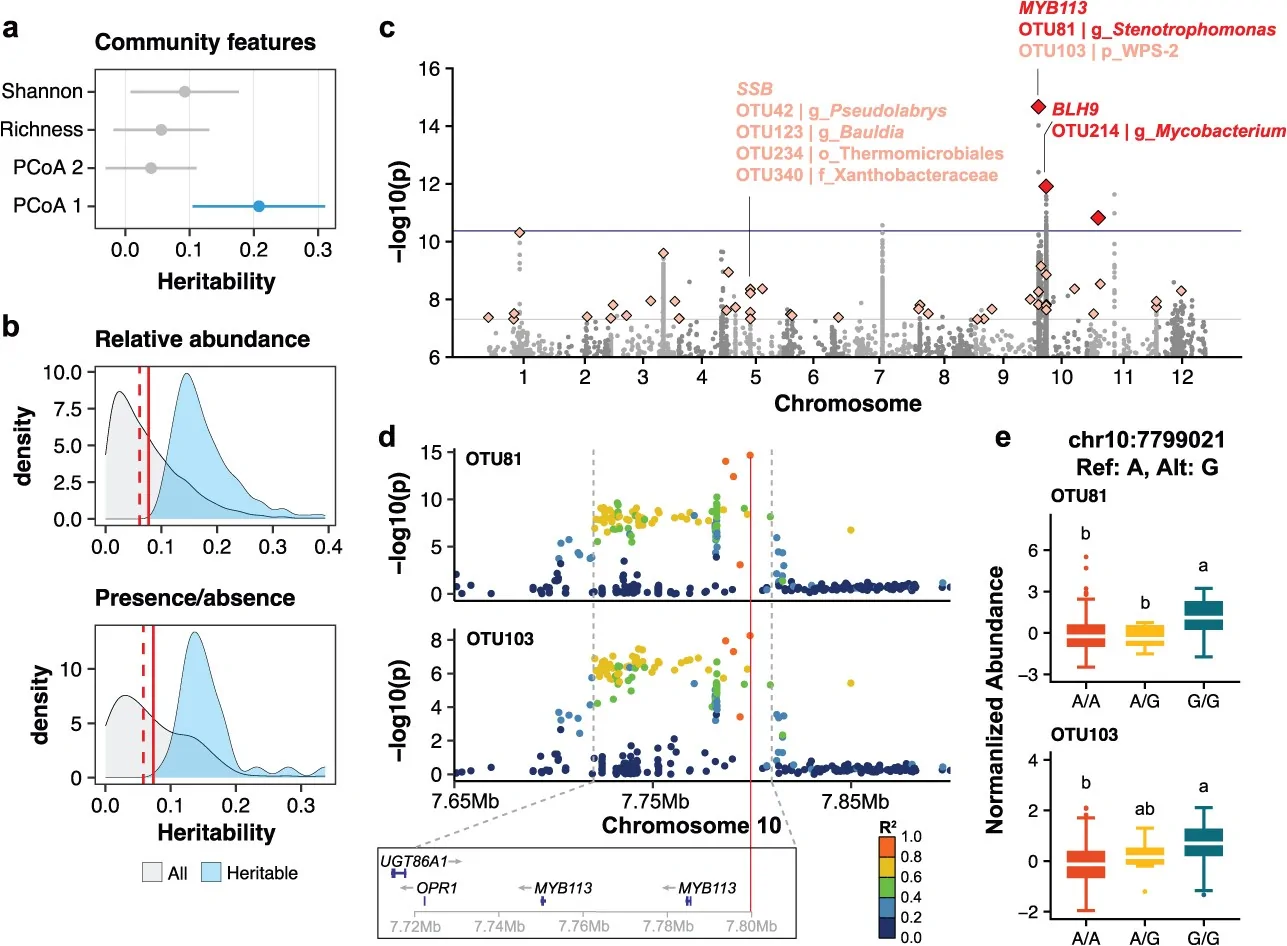
Host genetic control of rhizosphere microbiome in eggplant. (a) Heritability of eggplant rhizosphere microbial community features. Blue indicates significantly heritable community features [likelihood ratio test, *P_LRT_* < .05]. (b) Distribution of eggplant rhizosphere microbial heritability at different prevalence levels. Dashed and solid lines indicate the median and mean heritability of all OTUs, respectively. (c) Manhattan plot showing genome-wide association analysis results for core OTUs. The *y*-axis represents –log_10_ transformed *P*-value and the *x*-axis represents genomic positions of SNPs. Significance thresholds for study-wide (*P* < .05/3689651/315 = 4.30 × 10^−11^) and genome-wide (*P* < 5 × 10^−8^) levels are shown as blue and gray horizontal lines, respectively. Lead SNP achieving study-wide or genome-wide significance are marked with diamond symbols. (d) Fine-scale association mapping of OTU81 and OTU103 within the genomic regions surrounding the lead SNP *chr10:7799021*. (e) Genotype effect analysis for SNP *chr10:7799021*. Box plots show the impact of different genotypes on microbial abundance.

To investigate how eggplant genetic diversity influences rhizosphere microbial communities, we compared the genomic relationship matrix (GRM) and microbial relationship matrix (MRM) across 432 eggplant cultivars, revealing significant correlation between host genotype and microbiome (Supplementary Fig. 4a; *r* = 0.05, *P* < .001, Mantel’s test). We first evaluated the heritability (*h^2^*) of rhizosphere microbial community features, which reflect the overall properties of the microbiome. Only PCoA1 exhibited significant heritability (Fig. 3a; *h^2^* = 0.21, *P_LRT_* < .05). Given that PCoA1 accounted for the largest proportion of community variation (14.8%), this suggests that the main variation pattern in rhizosphere microbial communities is under host genetic control. We subsequently estimated heritability for individual OTUs by analyzing relative abundance as continuous traits for OTUs present in ≥50% of samples (*n* = 4859), while treating lower prevalence OTUs (<50% prevalence, *n* = 232) as binary presence/absence traits. Among all tested OTUs, 11.4% (533/4859) of continuous traits (relative abundance) and 13.4% (31/232) of binary traits (presence/absence) showed significant heritability (*P_LRT_* < .05), demonstrating that genetic control extends are not prevalence-dependent (Fig. 3b, Supplementary Table 6). However, OTUs with higher prevalence (Pearson’s *r* = 0.13, *P* < .001) or greater relative abundance (Pearson’s *r* = 0.18, *P* < .001) tended to exhibit increased heritability (Supplementary Fig. 4b). Heritability of significantly heritable OTUs (*P_LRT_* < .05) ranged from 0.09 to 0.39, significantly higher than those of nonheritable OTUs (Supplementary Fig. 5a). Among these heritable OTUs, OTU83 (*Pseudomonas*) exhibited the highest heritability (0.39, *P_LRT_* = 1.53e–4), while OTU5464 (*Pseudolabrys*) showed the lowest heritability (0.09, *P_LRT_* = 0.042; Supplementary Fig. 5b). Notably, the proportion of Rhizobiales was significantly higher among heritable OTUs compared to nonheritable OTUs (Supplementary Fig. 5c; Fisher’s exact test, *P* < .05).

Core microbiome taxa exhibited significantly higher heritability than noncore taxa (Supplementary Fig. 6a). Consistently, heritable OTUs were significantly enriched in the core microbiome: 23.17% of core OTUs (73/315; Fig. 2e) were heritable compared to only 10.69% of noncore OTUs (511/4776) (Fisher’s exact test, *P* < .05; Supplementary Fig. 6b). These heritable core taxa not only played important roles in the community, but also experienced stronger host genetic regulation, with heritability ranged from 0.10 to 0.39. Among them, the highest heritability was observed in OTU30, belonging to class AD3 within Chloroflexi (0.39, *P_LRT_* = 2.15e–4). In contrast, the lowest heritability was found in OTU82 from the genus *Granulicella* (0.10, *P_LRT_* = 0.015; Supplementary Fig. 5bb). Overall, these findings indicate that host genetics has widespread effects on the rhizosphere microbiome, but core and specific taxa are subject to stronger genetic regulation.

### GWAS identified genetic variants associated with eggplant core microbiome

**Figure 4:**
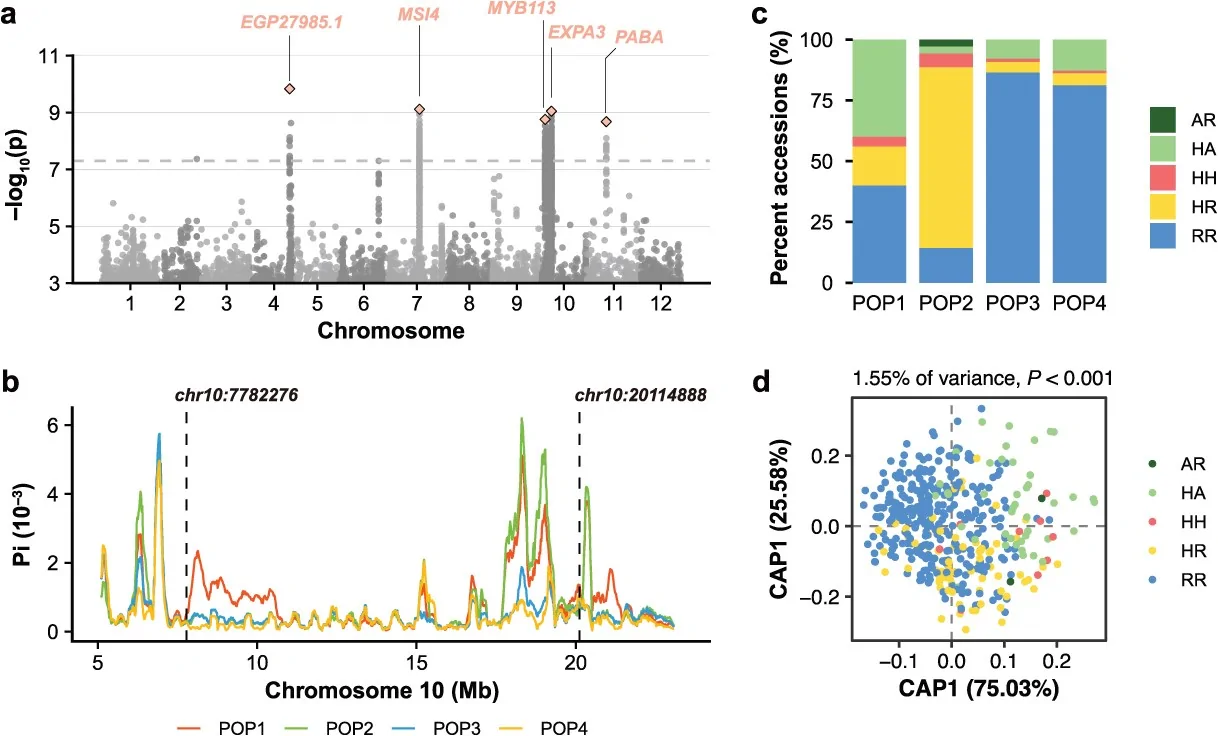
Allelic combinations of microbial community structure. (a) Manhattan plot of GWAS for PCoA2 of rhizosphere microbial communities. Horizontal dashed line indicates the genome-wide significance threshold (*P* < 5 × 10^−8^). Diamond symbols mark lead SNPs on chromosomes 4, 7, 10, and 11. Candidate genes near lead SNPs are labeled. (b) Nucleotide diversity (*π*) distribution on chromosome 10 across four populations (POP1–POP4). Vertical dashed lines indicate the positions of two lead SNPs *chr10:7782276* and *chr10:20114888*. (c) Distribution frequencies of allelic combinations at two lead SNP loci *chr10:7782276* and *chr10:20114888* across four populations. R indicates the reference allele, H indicates the heterozygous state, and A indicates the alternative allele. (d) Constrained analysis of principal coordinates (CAP) showing the relationship between allelic combinations and microbial community structure.

To further elucidate the genetic loci controlling rhizosphere microbial communities of eggplant, we conducted an mGWAS on 315 core OTUs. A total of 1235 genome-wide significant SNPs (*P* < 5 × 10^−8^) were identified (Fig. 3c and Supplementary Table 7), which were associated with 46 OTUs (Supplementary Fig. 7a). To explore the potential biological functions of significantly associated loci, we extracted all genes within ±1.06 Mb of the 1235 significant SNPs, a window corresponding to the LD decay distance, identifying 4038 genes. Kyoto Encyclopedia of Genes and Genomes (KEGG) enrichment analysis revealed substantial enrichment in primary and secondary metabolic pathways related to plant–microbe interactions (adjusted *P* < .05; Supplementary Fig. 7b), including anthocyanin biosynthesis, benzoxazinoid biosynthesis, and lipid metabolism. These findings suggest that host genetic variation influences rhizosphere microbial community composition through coordinated regulation of plant metabolism. (Fig. 4a; *P* < 5 × 10^−8^). Using a more stringent study-wide significance threshold of *P* < 4.30 × 10^−11^ (Bonferroni correction, 0.05/3689651 SNPs/315 OTUs), the strongest association was observed between OTU81 (*Stenotrophomonas*) and SNP *chr10:7799021* (Fig. 3c; *P* = 2.16 × 10^−15^). The candidate gene MYB113, located near SNP chr10:7799021, encodes a transcription factor that regulates anthocyanin biosynthesis across multiple plant species and functions critically in abiotic stress responses [23, 24]. The second most significant association was between SNP chr10:19786889 and OTU214 (*Mycobacterium*) (Fig. 3c and Supplementary Fig. 8a and b; *P* = 1.24 × 10^−12^), with this locus positioned near BLH9, which encodes a homeodomain transcription factor governing plant organ morphogenesis (such as leaves, stems, and roots), meristem maintenance, and environmental adaptation [25, 26].

We observed several genomic loci exhibited pleiotropic effects, with individual SNPs associated with two or more OTUs. For example, the lead SNP *chr10:7799021* was shared between OTU81 (*Stenotrophomonas*, *P* = 2.16 × 10^−15^) and OTU103 (WPS-2, *P* = 5.48 × 10^−9^; Fig. 3d, e). Furthermore, we found that taxonomically related microbes shared the same genetic loci. The SNP *chr5:34126740* exhibited simultaneous associations with four core rhizosphere microbial taxa [OTU42 (*Pseudolabrys*), OTU123 (*Bauldia*), OTU234 (Thermomicrobiales), and OTU340 (Xanthobacteraceae)], three of which belong to the order Rhizobiales (Supplementary Fig. 8c and d). To assess the specificity of this locus for Rhizobiales regulation, we examined association strengths between SNP chr5:34126740 and all 315 core OTUs. Rhizobiales OTUs (*n* = 21) demonstrated significantly stronger association signals compared to non-Rhizobiales OTUs (*n* = 294), with distinct –log₁₀ *P-*value distribution patterns (Supplementary Fig. 8e).

### Microbial community structure varies with different allelic combinations

To further investigate the association between eggplant genetics and the overall structure of rhizosphere microbial communities, we used the first two principal coordinate axes (PCoA1 and PCoA2) of microbial community as quantitative traits for GWAS, as these axes capture specific community-level variation across multiple microbial taxa. Although PCoA1 exhibited relatively high heritability, no genome-wide significant association loci were detected, suggesting that the community variation reflected by PCoA1 is likely controlled by numerous small-effect loci. PCoA2 exhibited significant association peaks on chromosomes 4, 7, 10, and 11 (Fig. 4a and Supplementary Fig. 9; *P* < 5 × 10^−8^). Comparison with mGWAS results at the individual OTU level revealed that multiple OTUs showed consistent associations at two signal peaks on chromosome 10 (Supplementary Fig. 10a). These OTUs primarily comprised Actinobacteria (6/9), WPS-2 (1/9), and Proteobacteria (1/9), further supporting the reliability of our genetic association analysis and suggesting that these microbial taxa are major contributors to the community differentiation represented by PCoA2. The lead SNPs at these two peaks were *chr10:20114888* (*P* = 8.9 × 10^−10^) and *chr10:7782276* (*P* = 1.7 × 10^−9^), located near candidate genes *MYB113* and *EXPA3*, respectively. Among them, *EXPA3* encodes α-expansin, a key regulatory protein involved in cell wall loosening during plant growth and plays a crucial role in root development processes [27].

Previous studies proposed that eggplant originated from the Southeast Asian domestication center and subsequently spread worldwide [28]. This domestication and dispersal pathway highly corresponds with our population differentiation results based on genetic structure analysis, with each subgroup exhibiting distinct geographical distribution patterns. Based on this, we used mGWAS results to further investigate whether microbial communities exhibit coevolutionary relationships with the host genome. We performed selective sweep analysis on the significantly associated regions of chromosome 10 and found that both lead SNPs were located within regions under selection (Fig. 4b and Supplementary Fig. 10b). Further analysis of the allelic combinations at these two SNP loci identified five distinct genotypic combinations (RR, HR, HH, HA, AR). The allelic frequency distribution showed strong subpopulation-specific patterns (Fig. 4c). In POP1, RR, and HA genotypes, each accounted for 40.00%. The European accession-dominated subpopulation (POP2) exhibited a markedly different pattern, with HR type reaching as high as 74.29%. In contrast, the Chinese accession-dominated subpopulations (POP3 and POP4) predominantly displayed the reference allelic combination (RR type) at these two loci, accounting for 86.40% and 81.19%, respectively. Notably, different allelic combinations significantly influenced microbial community structure (Fig. 4d). In conclusion, genetic loci associated with rhizosphere microbial community structure underwent strong directional selection during eggplant adaptation to different geographical environments, providing support for host–microbe coadaptive evolution.

## Discussion

Plant are not passive recipients of associated microorganisms [29]. Although stochastic events such as microbial interactions and environmental factors influence microbial assembly [30, 31], the genetic regulation-mediated ‘active management’ by plants plays a fundamental role in forming a beneficial microbiome that supports plant growth and health [32]. Therefore, systematically elucidating the molecular mechanisms by which plants precisely select and directionally recruit beneficial microbes represents a core scientific question for revealing the coevolutionary patterns between hosts and microbes. In this study, we performed the large-scale mGWAS in eggplant, integrating SNP datasets and rhizosphere microbiome profiles from 432 germplasm cultivars. Our findings revealed that core microbes serve as primary targets of host genetic regulation, and we identified several candidate genes potentially involved in regulating rhizosphere microbial community structure and composition. These results provide critical genetic insights into the precise regulation of microbiomes in crops and establish a theoretical framework for microbiome-assisted breeding strategies in eggplant.

Previous study has revealed that host genetic effects on associated microbiome are predominantly mediated by a small number of hub OTUs, which subsequently cascade through microbial networks to influence the entire community [33]. Our study further proved that such regulatory pattern exists ubiquitously in different host–microbe systems. More specifically, we found that the core microbes had a considerable enrichment of heritable taxa. Core microbial communities represent a small subset that persist stably within the ecosystem and perform critical functions. These microorganisms are very important for maintaining the complex microbial network system [34], mediating belowground nutrient cycling [35], and ensuring functional stability of soil microbiomes [36]. Accordingly, we conducted mGWAS to investigate the host genetic basis underlying the core rhizosphere microbiome in eggplant. Through this analysis, it was found that there were two important association peaks on chromosome 10, with *MYB113* and *BLH9* identified as the primary candidate genes. *MYB113* showed strong associations with an OTU classified as *Stenotrophomonas* and is a key regulator of anthocyanin biosynthesis [23, 24]. Plant anthocyanins as a subgroup of flavonoid secondary metabolites help plants gain stress tolerance by being potent antioxidant and UV protectants [37]. *Stenotrophomonas* is increasingly recognized as a plant growth-promoting bacterium, and some strains can produce various phytohormones, including auxin (IAA), gibberellin (GA), and abscisic acid (ABA), to enhance plant growth [38, 39]. Phytohormones play important regulators of anthocyanin biosynthesis. IAA and GA primarily function as repressors, whereas cytokinin (CK), ABA, ethylene (ETH), and jasmonic acid (JA) are mostly positive modulators [40]. Notably, exogenous ABA application has been shown to strongly induce MYB113 expression [41]. The *BLH9* gene is a member of the ubiquitous BEL1-like homeodomain (BLH) protein family in plants, with functions primarily related to plant development, regulating organ morphogenesis and tissue differentiation processes across multiple species [25, 26]. Our study identified a genetic locus significantly associated with an OTU from *Mycobacterium* located near the BLH9. Previous studies have demonstrated that various mycobacterial species can promote root development and plant growth through indole-3-acetic acid synthesis [42]. This functional coupling suggests that the observed BLH9-Mycobacterium association provides important insights into the microbial modulation of eggplant growth and development.

Heritability assessment can help us understand which microbial taxa are subject to host genetic control, serving as potential targets for plant–microbe interaction research [43]. The heritability of significantly heritable taxa in the eggplant rhizosphere microbiota ranged from 0.09 to 0.39, consistent with findings from studies of animal gut and other plant-associated microbial communities [15, 44, 45], and generally lower than heritability levels of conventional host agronomic traits. The relatively low heritability of microbiota can be attributed to several key factors. First, microbial communities are inherently complex, involving intricate interaction networks among hundreds of species [46]. Second, the spatial heterogeneity of the rhizosphere microenvironment far exceeds the environmental pressures encountered by agronomic traits [47], and the rapid response of microbial communities to environmental changes further increases the difficulty of accurately estimating their heritability. Third, methodological limitations also contribute to the relatively low heritability of microbial taxa. Amplicon sequencing provides limited taxonomic resolution, whereas host recruitment of microbes may be specific at the species or even strain level [48]. In addition, heritability is typically estimated using relative abundance data, which may also lead to underestimation [49]. Finally, complex indirect plant–microbe interactions further complicate heritability estimation, as host genotypes may shape microbial communities through root architecture, root exudate, plant immune responses, or overall plant growth status [50]. We found that Rhizobiales showed particularly high heritability values. Many members of Rhizobiales are rhizobia species, whose highly specialized symbiotic relationships with leguminous plants represent a classic example of plant–microbe interactions [51, 52]. However, Rhizobiales also commonly occur as dominant bacterial groups in nonleguminous plants [53], where they promote plant growth through diverse mechanisms even without forming true root nodules. These mechanisms include synthesizing phytohormones to regulate plant physiological processes and secreting siderophores to enhance plant iron nutrition [54, 55]. This universal functional importance may have driven plant hosts to maintain sustained genetic selection for Rhizobiales. Furthermore, we identified SNP *chr5:34126740* was significantly associated with multiple Rhizobiales OTUs. The *SSB* gene (single-stranded DNA-binding protein), located near this variant, is critical for DNA replication, recombination, and repair within organelles [56, 57]. While current knowledge of plant *SSB* function remains limited, studies in Arabidopsis have revealed that the mitochondrial SSB1 participates in mtRNA splicing and modulates plant responses to ABA [58].

Our study classified 432 eggplant varieties into four subpopulations that correlated with their geographical origins, consistent with previous research [59]. This geographical–genetic association pattern reflects the genetic differentiation of eggplant shape by the combined effects of geographical environment and artificial selection during long-term domestication, while also supporting current hypotheses regarding the origin, domestication, and global dispersal routes of eggplant [18, 28]. Eggplant rhizosphere microbiomes showed subtle but significant differences among subpopulations, this phenomenon also documented in Arabidopsis and tea plant [60–62]. Notably, the key genetic loci controlling microbial community structure, chr10:7782276 and chr10:20114888, were both located within selective sweep regions, indicating that they underwent strong directional selection during eggplant geographic dispersal. Specifically, these two lead SNPs showed distinct population-specific allelic combinations and significantly affected eggplant rhizosphere microbial community structure. These loci likely shape specific rhizosphere microbiomes by regulating plant root development (*EXPA3*) [27] and secondary metabolism (*MYB113*). This finding emphasizes the coadaption between plants and microbes.

In summary, we reveal host genetic mechanisms underlying eggplant rhizosphere microbial communities through large-scale mGWAS and fill fundamental knowledge gaps pertaining to plant–microbe interactions. Together, these discoveries form a concept basis for microbiome-assisted breeding and sustainable agricultural development in eggplant. However, translating these findings into practical applications still faces several challenges and limitations. This study was conducted under greenhouse conditions, which facilitated the precise detection of statistical associations between host genotypes and microbial communities, but may not fully capture the complexity of natural ecosystems. Although these association analyses provide important clues, the observed relationships still require further confirmation through functional validation and mechanistic studies. In addition, 16S rRNA amplicon sequencing has limited taxonomic resolution and usually only can be annotated at the genus level, making it difficult to accurately infer the ecological functions of specific microbes based on genus-level taxonomic information. Future studies should integrate gene editing, metagenomics, root metabolomics, and microbial inoculation experiments, while also incorporating fungi and other microbial groups under more complex environmental conditions, to further elucidate the molecular mechanisms underlying candidate gene–microbiome interactions and facilitate the development of microbiome-assisted breeding strategies.

## Methods

### Plant materials and sampling

The eggplant germplasm resources used in this study were provided by the Vegetable Research Institute of Guangxi Academy of Agricultural Sciences. All materials belong to the cultivated species *S. melongena* L. and represent a global collection spanning 31 Chinese provinces, municipalities, and autonomous regions, as well as multiple countries across Asia, Europe, America, Oceania, and Africa. The Chinese materials were primarily sourced from Sichuan, Guangxi, Shandong, and Liaoning provinces, while international materials originated from Vietnam, France, the USA, Germany, and other countries (Supplementary Table 1).

All eggplant varieties were grown in sterilized substrate until the four-leaf stage, then transplanted to the greenhouse at the Lijian Scientific Research Base in Nanning, Guangxi Province, China (23°14′49.77″N, 108°3′26.16″E). Following transplanting, all accessions were planted under identical soil and environmental conditions in three replicate blocks, to minimize the influence of environmental heterogeneity. Three weeks after transplanting, three uniformly growing healthy plants were randomly selected from each accession within each block for sampling, with samples pooled to constitute one biological replicate. Young leaves were collected using sterile scissors from the top unexpanded leaves of each plant. For rhizosphere soil sampling, whole plants were carefully uprooted, and the roots were gently shaken by hand to remove loosely attached soil. The soil particles that remained tightly adhered to the root surface were then brushed off, thoroughly homogenized, and placed into a 2-ml centrifuge tube as one rhizosphere soil sample. Subsequently, 0.5 g of each sample was accurately weighed for microbial community analysis. All leaf and rhizosphere soil samples were stored at −80°C until DNA extraction.

### Whole-genome resequencing

Total DNA was extracted from eggplant leaves using the E.Z.N.A. Tissue DNA kit (Omega Bio-Tek) following the Tissue DNA-Spin Protocol. After the DNA samples were delivered, a quality control test was carried out on the specimens, and the qualified DNA (>3 μg; concentration >30 ng/μl; OD260/OD280 = 1.80–2.00) was used to do further study. For Illumina paired-end sequencing, at least 3 μg of genomic DNA from each sample was used for library construction. Paired-end libraries with an insert size of ~450 bp were prepared according to Illumina’s standard protocol and sequenced on the Illumina NovaSeq 6000 platform (150 bp × 2, Shanghai Biozeron Biotechnology Co., Ltd, Shanghai, China). The raw paired end reads were trimmed and quality controlled by Trimmomatic [63] (version 0.36, http://www.usadellab.org/cms/uploads/supplementary/Trimmomatic) to obtain high-quality clean reads. The 2019 published eggplant genome was used as the reference genome [64]. The clean sequencing reads are aligned to the reference genome sequence using BWA (http://bio-bwa.sourceforge.net/) software (bwa mem -k 32). Sequence Alignment Map (SAM) format files were imported into SAMtools (http://samtools.sourceforge.net/) for sorting and merging and into Picard (http://broadinstitute.github.io/picard/, version 1.92) to remove duplicated reads. The sequencing depth and coverage were calculated based on the alignments by custom perl scripts. The valid BAM file was used to detect SNPs by GATK (version 4.1.2.0) ‘HaplotypeCaller’ function (http://www.broadinstitute.org/gatk/). Followed variant call format (VCF) files were generated by quality filtering (VCFtools, VariantFiltration with parameters: QD < 2.0||FS > 60.0||MQ <40.0||SOR > 10.0). The annotation of detected SNPs can be performed by ANNOVAR (http://www.openbioinformatics.org/annovar/).

We retained only SNPs with a minor allele frequency (MAF) >0.05 and a missing rate <0.5 for subsequent analysis. LD decay was assessed using PopLDdecay (v3.42) to calculate allele correlation coefficients (r [2]) across the eggplant population. The LD decay distance was determined to be 130 kb, corresponding to the point where the average *r*^2^ value dropped to half of its maximum (Supplementary Fig. 1b). A neighbor-joining phylogenetic tree constructed using SNP data, by GCTA v1.94.1 to compute genetics distance (GRM) [65], then use FastTree v2.1.11 to construct NJ tree. In order to do principal component analysis within the peach population, we calculated the eigenvector decomposition of the matrix using the GCTA v1.94.1. The program Admixture was used to perform population structure analysis. It was based on the maximum likelihood method [66]. Before using Admixture, we used PLINK v1.9 to generate the needed map files. The input parameter K was changed from 2 to 9, representing the assumed groups of the simulated population in ancient times. The optimal number of genetic clusters (K) is conventionally selected by minimizing the cross-validation error (CV error). However, in our study, K = 4 was found to yield subpopulations that demonstrated the highest degree of consistency with geographical origins.

### 16S rRNA amplicon sequencing

Microbial DNA was extracted from rhizosphere soil samples using the E.Z.N.A.® Soil DNA Kit (Omega Bio-tek, Norcross, GA, USA) according to manufacturer’s protocols. The V4–V5 hypervariable region of the bacteria 16S ribosomal RNA gene were amplified by PCR (95°C for 2 min, followed by 25 cycles at 95°C for 30 s, 55°C for 30 s, and 72°C for 30 s and a final extension at 72°C for 5 min) using 515F/907R primers. Amplicons were separated by 2% agarose gels electrophoresis and purified using the AxyPrep DNA Gel Extraction Kit (Axygen Biosciences, Union City, CA, USA), then quantified with a Qubit®3.0 (Life Invitrogen). Amplicons were pooled in equal amounts to construct libraries, fragmented by ultrasonication, and then subjected to paired-end 300-bp (PE300) high-throughput sequencing on the Illumina MiSeq platform (Shanghai BIOZERON Biotech. Co., Ltd).

The raw sequencing data underwent strict quality control and data cleaning, including removal of bases with quality scores <20, filtering of sequences shorter than 50 bp, and sequence merging (with a maximum mismatch rate of 0.2 and minimum overlap length of 10 bp). After identifying and removing chimeric sequences using UCHIME software, sequences were clustered into operational taxonomic units (OTUs) at 97% similarity using the UPARSE (version 7.1, http://drive5.com/uparse/) algorithm. Finally, OTU representative sequences were taxonomically annotated using the RDP classifier’s (http://rdp.cme.msu.edu/) Bayesian algorithm against the Silva (SSU138) 16S rRNA database [67] (confidence threshold of 80%), yielding microbial community composition and diversity information for each sample at different taxonomic levels.

### Rhizosphere core microbial taxa

We defined OTUs present in all samples (100% prevalence) as the rhizosphere core microbial taxa. Considering that prevalence-based screening strategies may overlook functionally and ecologically important taxa with low abundance below detection thresholds in certain samples [68], we further constructed microbial co-occurrence network based on relative abundance data. Spearman correlation analysis was conducted with Bonferroni multiple testing correction, establishing a correlation coefficient matrix with thresholds of *r* > 0.6 and *P* < .05. Co-occurrence networks were subsequently visualized in R using the *igraph* (v2.1.4) package. Network nodes were classified into four categories based on within-module connectivity (Zi) and among-module connectivity (Pi) [69]: Module hubs (highly connected to many nodes in their own modules, Zi >2.5 and Pi <0.62), Connectors (highly linked to several modules, Zi <2.5 and Pi >0.62), Network hubs (high connectivity throughout the entire network, Zi >2.5 and Pi >0.62), and Peripherals (low connectivity both within and between modules, Zi <2.5 and Pi <0.62). All node types except Peripherals are considered keystone nodes that play essential roles in maintaining microbial network stability [22]. These keystone OTUs were therefore included as components of the rhizosphere core microbial taxa.

### Evaluation of heritability

We classified OTUs based on prevalence: those present in >50% of samples were analyzed as quantitative traits using their relative abundance, while those present in ≤50% of samples were treated as binary traits (presence/absence) [70]. Specifically, relative abundance >0 were coded as 1 (presence), and those equal to 0 were coded as 0 (absence). In addition, we produced a centered log ratio (CLR)-transformation for quantitative traits data to achieve normal distribution for heritability assessment. We preformed genome-based restricted maximum likelihood analysis [65], as implemented in the GCTA v1.94.1, to estimate the proportion of variance in rhizosphere microbial community characteristics explained by all SNPs. These microbial characteristics encompassed two alpha-diversity indices, the first two PCoA axes, 4859 quantitative traits, and 232 binary traits. Meanwhile, the top 10 host genetic principal components (PCs) were included as covariates in the model to control for population stratification effects. A likelihood ratio test (LRT) was used to assess the significance of the heritability estimates for microbial traits (*P_LRT_* < 0.05).

### Construction of microbial relationship matrix

To explore the effect of host genetic variation on rhizosphere microbial community, we performed a Mantel test to assess the correlation between the MRM and the previously constructed GRM using Pearson correlation (9999 permutations). The MRM was computed as follows [70]:


(1) \begin{equation*} {m}_{ij}=\frac{1}{N}\sum_{O=1}^N\frac{\left({O}_{io}-\overline{O_o}\right)\left({O}_{jo}-\overline{O_o}\right)}{\sigma_o^2} \end{equation*}

In the above equation, ${m}_{ij}$ is the microbial relationship between assessed samples *i* and *j*; ${O}_{io}$ and ${O}_{jo}$ is the relative abundance of OTU*_O_* in samples *i* and *j*, respectively; ${\sigma}_o^2$ is the variance of relative abundance of OTU*_O_*; and *N* is the total number of OTUs used to assess intersample microbial correlations.

### Microbial genome-wide association studies

To establish mixed linear models (MLM) for identifying SNPs significantly associated with microbial community characteristics (the first two PCoA axes) and 315 core OTUs, we employed GEMMA software [71] (https://github.com/genetics-statistics/GEMMA) with the ‘-lmm 1’ parameter to construct univariate mixed linear models for detecting significant SNPs. The kinship matrix and the top 10 host genetic principal components were incorporated as covariates to control for the effects of eggplant population stratification on association results. For the rhizosphere 315 core OTUs (all present in >50% of samples and treated as quantitative traits), their relative abundance data were subjected to CLR transformation before being used as phenotypic inputs for GWAS analysis. Significance thresholds were set as follows: genome-wide significance threshold at *P* < 5 × 10^−8^, and study-wide significance threshold at *P* < 4.30 × 10^−11^ (Bonferroni correction, 0.05/3689651 SNPs/315 OTUs).

To identify candidate genes from the mGWAS analysis, LD blocks were constructed using PLINK software (v1.9). Genes that were located within or partially overlapped with LD blocks containing significant SNPs were considered as candidate genes associated with the traits. When no genes were found to overlap within the LD blocks, or when significant SNPs were not located within any LD blocks, the nearest upstream and downstream genes flanking the significant SNPs were identified as potential candidate genes. Using the reference eggplant gene set as the background [64], the identified candidate genes were subjected to KEGG pathway enrichment analysis using TBtools-II (v2.376) [72, 73].

### Statistical analysis

Microbial community alpha diversity was calculated using the *vegan* (v2.6–8) package. PCoA and PERMANOVA were performed using the *vegan* (v2.6–8) and *ape* (v5.8) packages. For statistical analyses, multiple group comparisons (≥3 groups) were conducted using one-way ANOVA with *post hoc* multiple comparisons performed by Tukey’s HSD test, while pairwise comparisons between two groups employed Wilcoxon rank-sum tests. The significance level for all statistical tests was set at *P* < .05.

## Supplementary Material

Web_Material_uhag163

## Data Availability

The raw 16S rRNA gene amplicon sequencing data and whole-genome sequencing data generated in this study have been deposited in the NCBI Sequence Read Archive (SRA) under BioProject accession numbers PRJNA1377905 and PRJNA1155721, respectively.
