# Supplementary material for: Genetic dissection of the eggplant rhizosphere microbiome enables microbiome-assisted breeding
Source: Hortic Res. 2026 Apr 23;13(9):uhag163. doi: 10.1093/hr/uhag163 (PMC13523853; doi:10.1093/hr/uhag163)
Supplement: Supplementary file 1 [file web_material_uhag163.zip › Supplementary Figures.docx]

Supplementary information

**Supplementary Fig.1 The genetic diversity of 432 eggplant varieties. a,** Distribution of SNPs along chromosomes. **b,** Genome-wide linkage disequilibrium (LD) decay. The X-axis represents genetic distance between SNPs (kb), and the Y-axis represents LD values (R²). Horizontal and vertical dashed lines indicate half LD and LD decay distance, respectively. LD decays to its half-decay distance at 1.06 Mb.

**Supplementary Fig.2 Differences in rhizosphere microbial community composition and alpha diversity among eggplant subpopulations. a,** Rhizosphere microbial composition at phylum level. **b,** Comparison of rhizosphere microbial richness (left) and shannon (right) diversity index across eggplant subpopulations. Different letters indicate significant differences among subpopulations according to one-way ANOVA followed by the LSD post hoc test (p < 0.05).

**Supplementary Fig.3 Definition of the eggplant rhizosphere core microbiome. a,** Relative abundance and number of OTUs at different prevalence levels in the eggplant rhizosphere. **b,** Overview of the co-occurrence networks. Each node represents a bacterial OTU and is colored by phylum. The size of the node is proportional to its degree. **c,** *Zi*-*Pi* plots to identify putative keystone OTUs within the rhizosphere networks. Threshold values of *Zi* and *Pi* for classification are 2.5 and 0.62, respectively.

**Supplementary Fig.4 Host genetic control of rhizosphere microbiome and heritability estimates. a,** Correlation between host genetic relationship matrix and microbial relationship matrix. **b,** Pearson correlation of heritability with OTU prevalence (left) and relative abundance (right).

**Supplementary Fig.5 Heritability of rhizosphere bacterial OTUs. a,** Comparison of heritability estimates between non-heritable (*P_LRT_* ≥ 0.05) and heritable (*P_LRT_* < 0.05) OTUs (Wilcoxon rank-sum test, *p* < 0.001). **b,** Distribution of heritability estimates for the top 20 (highest *h²*) and bottom 20 (lowest *h²*) heritable OTUs, ranked by *h²* values. Red labels on the y-axis indicate core microbiome taxa. **c,** Proportional composition of the top 15 bacterial orders across all, non-heritable (*P_LRT_* ≥ 0.05), and heritable OTUs (*P_LRT_* < 0.05). Asterisks indicate orders with significantly different proportions between heritable and non-heritable fractions, determined by Fisher's exact test (*p* < 0.05).

**Supplementary Fig.6 Heritability of core microbiome (a) and the proportions of heritable versus non-heritable OTUs within the core microbiome (b).** Statistical significance was assessed using Wilcoxon rank-sum test.

**Supplementary Fig.7 Genome-wide association study results and functional enrichment analysis. a,** Number of OTUs with significant associations (*P* < 5 × 10⁻⁸) at the phylum level. **b,** KEGG enrichment results of genes near significantly associated loci. Genes located within the LD decay distance (±1.06 Mb) of the 1,235 significant SNPs were extracted for enrichment analysis.

**Supplementary Fig.8 Fine-scale mapping and genotypic effect analysis of key SNPs. a,** Fine-scale association mapping of OTU214 within the genomic regions surrounding the lead SNP chr10:19786889. Points in different colors represent different linkage disequilibrium (LD) intensities. Gene annotation information for these regions is displayed below. **b,** Genotype effect analysis for SNP chr10:19786889. **c,** The lead SNP chr5:34126740 was shared among multiple OTUs within the order Rhizobiales. Fine-scale association mapping of four core OTUs within the genomic regions surrounding the lead SNP chr5:34126740. Points in different colors represent different linkage disequilibrium (LD) intensities. Gene annotation information for these regions is displayed below. **d,** Genotype effect analysis for SNP chr5:34126740. Box plots show the impact of different genotypes on microbial abundance. **e,** Association strength comparison between Rhizobiales and other bacterial orders at SNP chr5:34126740, Statistical significance was assessed using Wilcoxon rank-sum test.

**Supplementary Fig.9 GWAS analysis of PCoA1 and QQ plot. a,** Manhattan plot of genome-wide association study (GWAS) for PCoA1 of rhizosphere microbial communities. Horizontal dashed line indicates the genome-wide significance threshold (*P* < 5 × 10⁻⁸). **b,** QQ plot for PCoA1 GWAS results. **c,** QQ plot for PCoA2 GWAS results. The red line represents the expected theoretical distribution.

**Supplementary Fig.10 Genome-wide association analysis of multiple OTUs and population genetic differentiation. a,** Manhattan plot showing GWAS results of multiple OTUs associated with the same region as PCOA2. **b,** Genetic differentiation (*F_ST_*) distribution across chromosome 10 for six pairwise population comparisons. Vertical dashed lines indicate the positions of two lead SNPs *chr10:7782276* and *chr10:20114888*. Horizontal dashed lines represent the FST threshold, indicating the top 10% of genomic regions with high differentiation.
